# Management of RANKL-mediated Disorders With Denosumab in Children and Adolescents: A Global Expert Guidance Document

**DOI:** 10.1210/clinem/dgad657

**Published:** 2023-12-02

**Authors:** Joel A Vanderniet, Vivian Szymczuk, Wolfgang Högler, Signe S Beck-Nielsen, Suma Uday, Nadia Merchant, Janet L Crane, Leanne M Ward, Alison M Boyce, Craig F Munns

**Affiliations:** Sydney Medical School, Faculty of Medicine and Health, The University of Sydney and Institute of Endocrinology and Diabetes, The Children’s Hospital at Westmead, Sydney, NSW 2145, Australia; Metabolic Bone Disorders Unit, National Institute of Dental and Craniofacial Research, National Institutes of Health, Bethesda, MD 20814, USA; Department of Paediatrics and Adolescent Medicine, Johannes Kepler University Linz, Linz 4020, Austria; Centre for Rare Diseases, Aarhus University Hospital and Department of Clinical Medicine, Aarhus University, Aarhus N DK-8200, Denmark; Department of Endocrinology and Diabetes, Birmingham Women's and Children's Hospital and Institute of Metabolism and Systems Research, University of Birmingham, Birmingham B15 2TG, UK; Division of Endocrinology and Diabetes, Children's National Hospital, Washington, DC 20010, USA; Department of Pediatrics and Department of Orthopedic Surgery, Johns Hopkins University, Baltimore, MD 21287, USA; Department of Pediatrics, University of Ottawa and Division of Endocrinology, Children's Hospital of Eastern Ontario, Ottawa, Ontario K1H 8L1, Canada; Metabolic Bone Disorders Unit, National Institute of Dental and Craniofacial Research, National Institutes of Health, Bethesda, MD 20814, USA; Child Health Research Centre and Mayne Academy of Paediatrics, University of Queensland, Brisbane, QLD 4101, Australia

**Keywords:** giant cell tumor, aneurysmal bone cyst, fibrous dysplasia, rebound hypercalcemia, hypocalcemia, bone mineral density

## Abstract

**Context:**

Denosumab is an effective treatment for many receptor activator of nuclear factor kappa-B ligand (RANKL)-mediated disorders but there are potential safety considerations and limited data to guide its use in children and adolescents.

**Objective:**

This document seeks to summarize the evidence and provide expert opinion on safe and appropriate use of denosumab in pediatric RANKL-mediated disorders.

**Participants:**

Ten experts in pediatric bone and mineral medicine from 6 countries with experience in the use of denosumab participated in the creation of this document.

**Evidence:**

Data were sourced from the published literature, primarily consisting of case reports/series and review articles because of the lack of higher level evidence. Expert opinion of the authors was used substantially when no published data were available.

**Conclusion:**

Denosumab is an effective treatment for RANKL-mediated disorders in children and adolescents but is often not curative and, in some cases, is best used in conjunction with surgical or other medical treatments. Careful multidisciplinary planning is required to define the goals of treatment and expert oversight needed to manage the risk of mineral abnormalities. Substantive, collaborative research efforts are needed to determine optimal treatment regimens and minimize risks.

##  

A meeting was held during the 2022 International Conference on Children's Bone Health in Dublin, Ireland, to discuss the development of a shared approach to management of receptor activator of nuclear factor kappa-B ligand (RANKL)-mediated disorders with denosumab in children and adolescents and to address safety concerns. Twenty-eight experts in pediatric bone and mineral medicine from all continents were present. There was agreement that denosumab is an effective treatment for rare, disabling RANKL-mediated disorders in children, such as giant cell-rich bone tumors and fibrous dysplasia (FD), and potential treatment for others, such as juvenile Paget disease (JPD), for which other effective treatments are lacking. Denosumab treatment can, in some RANKL-mediated disorders, be discontinued when the treatment goal has been met, but in others needs tapering to the minimally effective dose to maintain control of the target disorder. The associated risks, especially of rebound hypercalcemia, were considered significant and have led to recommendations against its use in children by some professional bodies (1). However, this categorical approach limits options for patients with surgically unresectable lesions for whom denosumab may provide benefit. There is therefore a need for expert recommendations on the most effective way to manage the risk:benefit profile of denosumab treatment in pediatric specialist care. The group expressed a need for continued international collaboration to refine these recommendations and encourage collaborative research to produce more data.

A writing group was formed with representatives from each global region with experience in the use of denosumab in children and adolescents and tasked with the authorship of this document, which is intended to inform decisions and planning by local teams, but not to be prescriptive. Because of the limited published data of denosumab therapy in pediatrics, only low-quality evidence from case reports and series was available to inform the recommendations made. All unreferenced recommendations are based on amalgamated expert opinion. Denosumab should only be prescribed to children and adolescents in centers of expertise in managing rare diseases, under the supervision of a pediatric endocrinologist with expertise in managing denosumab therapy and the potential mineral abnormalities. Regional collaboration and discussion are encouraged to inform local practice.

## RANKL-mediated Disorders

RANKL is expressed by osteogenic cells and induces osteoclast differentiation by binding to RANK on osteoclast precursors. Osteoprotegerin (OPG) is a decoy receptor produced by osteogenic cells to inhibit RANKL signaling. The balance between RANKL and OPG is critical to maintain skeletal homeostasis, which requires a precise balance of bone formation and resorption. This balance is disrupted in RANKL-mediated disorders, resulting in focal bone destruction or systemic unregulated bone turnover (2).

RANKL-mediated bone tumors include giant cell tumors of bone (GCTB), aneurysmal bone cysts (ABC), and central giant cell granulomas (CGCG) of the mandible and maxilla. Although separate entities with some differences in histomorphology, these tumors share a similar pathophysiology involving a neoplastic population of stromal cells that produce RANKL in an uncontrolled fashion. The result is osteoclast-like giant cells that form a lytic, multilocular lesion (3-6). They can be locally aggressive and destructive, but only GCTB has malignant potential (7). CGCG may be associated with Noonan syndrome, neurofibromatosis type 1, and cherubism (5), whereas a translocation causing upregulation of the *USP6* oncogene is implicated in the pathogenesis of primary ABC (8). Surgical management of RANKL-mediated bone tumors is often associated with significant morbidity, particularly for lesions of the face or axial skeleton, and recurrence rates are high after curettage or incomplete resection (5, 9-11). Calcitonin, intralesional corticosteroids, radiotherapy, and subcutaneous interferon have been used as primary or adjuvant treatment with variable efficacy and generally high recurrence rates (5, 9-11). Bisphosphonate therapy has shown efficacy in some case reports (12-17) and requires further study to understand efficacy and recurrence rates.

Juvenile xanthogranuloma is a non-Langerhans cell histiocytosis most commonly affecting the skin but also occurring extracutaneously, including very rarely in the bone. Osseous lesions are slow-growing benign lesions containing histiocytes, spindle cells, and osteoclast-like giant cells (18, 19). Most nonosseous lesions either resolve spontaneously or are treated with complete surgical excision. However, reported spinal lesions have not resolved spontaneously and have not been amenable to complete excision (19, 20). Radiotherapy and chemotherapy have been used as adjuvants to subtotal resection in some lesions (21).

FD is a complex mosaic skeletal disorder that arises because of gain-of-function variants in the cAMP-regulating protein Gα_s_ (22). Constitutive receptor signaling alters osteoprogenitor cell differentiation, and proliferation of these cells results in the formation of discrete fibro-osseous lesions. These expansile and fragile lesions cause morbidity because of fractures, deformity, pain, and, if the craniofacial skeleton is involved, vision and hearing impairment. Radiographically, lesions have a characteristic “ground glass appearance” (23) with histology demonstrating abnormal woven bone with fibrotic stroma and prominent osteoclastogenesis (24). FD tissue is in a high turnover state with increased RANKL expression by osteoprogenitor cells (25). Inhibition of the RANKL pathway has been demonstrated to arrest lesion progression in murine models and to reduce lesion activity by promoting osteoprogenitor cell maturation and lesion mineralization in adults (26, 27). Current treatment of FD centers around surgical intervention to correct deformities and fractures, although outcomes are often unsatisfactory because postoperative regrowth is frequent in the still-growing skeleton (28, 29). Thus, corrective surgery of bone overgrowth in craniofacial FD is not recommended in the absence of functional deficits until skeletal growth is complete (30). Bisphosphonates have not shown to directly impact lesion progression or activity but can improve FD-related bone pain (31, 32).

JPD is a rare autosomal-recessive disorder arising because of inactivating variants in *TNFRSF11B*, which encodes OPG (33). OPG deficiency results in unopposed RANK signaling and promotes osteoclastogenesis. Generalized rapid bone turnover causes bone expansion, resulting in progressive skeletal deformity, fractures, pain, and skull enlargement, along with extraskeletal manifestations (34). Patients have significantly elevated serum alkaline phosphatase activity and elevation of other bone turnover markers. Radiographically, bone demonstrates diffuse hyperostosis, osteosclerosis, and marked cortical thickening (35), with histology demonstrating abnormal and fragile woven bone with numerous osteoclasts (33). JPD is generally treated with bisphosphonates and palliative surgical interventions (34, 36). Recombinant OPG has been tried but is not available for routine clinical use (37).

## Denosumab and Its Use in Children and Adolescents

Denosumab is a fully human monoclonal antibody to RANKL and leads to potent inhibition of bone resorption. It halts osteoclastic activity and reforms mineralized bone in RANKL-mediated bone tumors, demonstrated in large prospective studies in adults (9, 38, 39). Similar efficacy in other RANKL-mediated disorders has been reported in case reports and series (2, 27, 32, 40).

**Figure 1.**
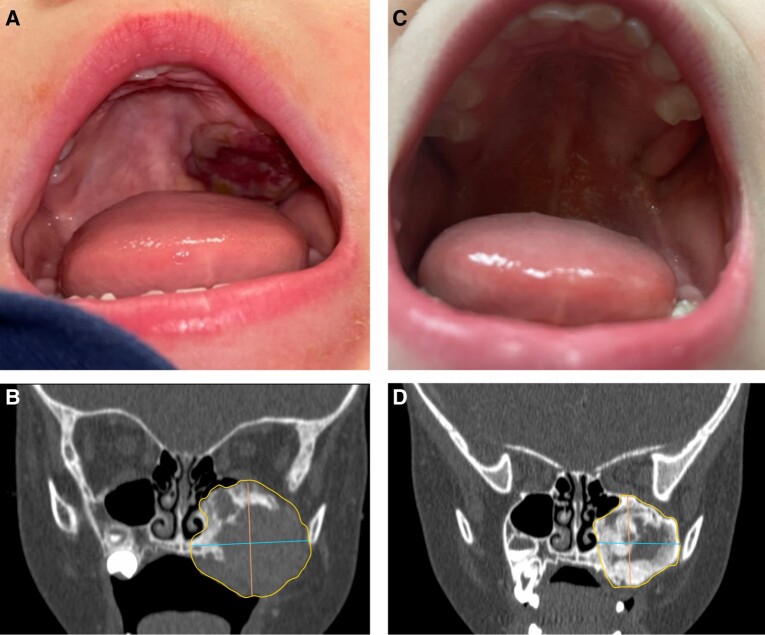
Representative images of denosumab treatment in central giant cell granuloma. The left-hand panels show baseline images from a 2-year-old boy. This includes a photograph showing an expansile palatal mass (A) and computed tomography demonstrating an expansile lytic lesion of the maxilla with thinned cortices and areas of cortical destruction (B). The right-hand panels show response after denosumab treatment, including regression of the palatal mass (C) and progressive peripheral sclerosis with increased radiodensity (D).

**Figure 2.**
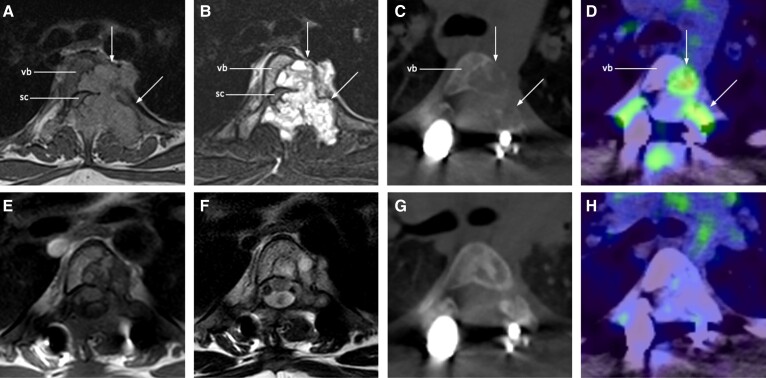
Representative images of denosumab treatment in spinal ABC. MRI T1-weighted (A) and STIR (B) images show a lobulated, expansile lesion (arrows) of T4 vertebral body (vb) and posterior elements with spinal cord (sc) stenosis, before partial resection and spinal stabilization with rods and bone graft. Postsurgery but before denosumab, the remaining ABC is seen as a lytic lesion in the left T4 vertebral body and pedicle on low-dose CT (C) with FDG avidity on PET (D). After 6 months of denosumab treatment, MRI T1-weighted (E) and T2-weighted (F) images show reduced signal intensity, noting metallic artifact from spinal rods reduces quality of images; peripheral sclerosis with increased radiodensity is seen on low-dose CT (G) and resolution of FDG-avidity on PET (H). ABC, aneurysmal bone cyst; CT, computed tomography; FDG, fluorodeoxyglucose; MRI, magnetic resonance imaging; PET, positron emission tomography; STIR, short tau inversion recovery.

**Figure 3.**
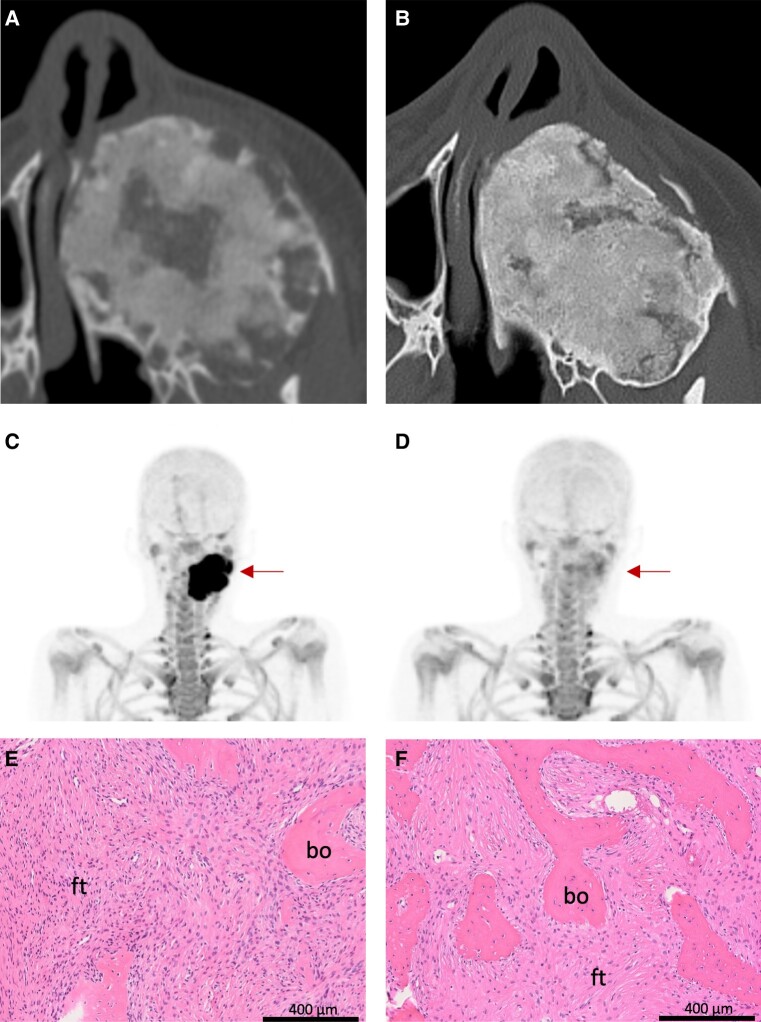
Representative images of denosumab treatment response in fibrous dysplasia (FD). The top panels show computed tomography images of a child with aggressive craniofacial FD. (A) Baseline scan at age 10 years shows an expansile lesion in the left maxilla. Note the heterogeneous appearance, with areas of radiolucency interspersed with more typically appearing “ground glass” radiopaque regions. (B) Two years after denosumab treatment the lesion has shown no further expansion and has developed a more homogenous, radiodense appearance. Based on her clinical response, the decision was made to discontinue denosumab treatment. The middle panels show ^18^F-NaF PET/CT imaging from this patient 3 years later (age 19 years), at which point FD expansion and pain had returned. (C) The maxillary lesion shows high tracer avidity (arrow), consistent with reactivation of FD lesion activity. The decision was made to restart denosumab treatment. (D) Six months later, the tracer avidity has declined to near-physiologic levels, correlating with improvement in her symptoms. The bottom panels show FD tissue from an adult patient pre- (E) and post- (F) denosumab treatment. Note the overall reduction in cellularity of the fibrous tissue (ft) and increased bone formation (bo) within the lesion.

There is limited experience globally with the use of denosumab in children and adolescents, given the rarity of RANKL-mediated disorders. Published data suggest that denosumab is highly effective in rapidly halting osteoclastic activity and expansion of focal lesions in RANKL-mediated bone tumors (Figs. 1 and 2) and FD (Fig. 3), with subsequent ossification during continued treatment (2, 10, 19, 27, 32, 38, 41-66). However, lesion reactivation rates after cessation of treatment are substantial, with some lesions requiring subsequent surgery to achieve a cure or long-term symptom relief, or otherwise long-term denosumab treatment.

Denosumab administered to a patient with JPD resulted in normalization of alkaline phosphatase levels and resolution of bone pain but was associated with severe disturbance of mineral homeostasis. Severe hypocalcemia developed rapidly and required prolonged IV calcium administration, so treatment was abandoned after the second dose (67). The patient also experienced hypercalcemia 7 weeks later. Although the potential benefits of denosumab in JPD are significant, a regimen with a favorable risk:benefit profile has not yet been established and would presumably require close monitoring and timely intervention to maintain eucalcemia.

Complete surgical resection results in the lowest recurrence rates in RANKL-mediated bone tumors (5, 11, 68, 69). Where this is not possible or desirable because of the associated morbidity, denosumab has been used either as primary therapy or as an adjuvant or neoadjuvant to a subtotal surgical approach (70). Data on the recurrence risk of GCTB after neoadjuvant denosumab and intralesional curettage have been conflicting, with some reports showing a higher recurrence rate and others not (71, 72). Nevertheless, this approach is increasingly being used for borderline or unresectable lesions to achieve mineralization and devascularization and improve operability (71-73).

There is a risk of hypocalcemia during treatment, but symptomatic hypocalcemia is typically preventable with adequate calcium and cholecalciferol supplementation (2, 9, 44-46, 49). Children with FD and high baseline bone turnover are at higher risk of hypocalcemia and typically require more vigorous calcium supplementation in combination with calcitriol or alfacalcidol. Hypophosphatemia can also occur during treatment, and occasionally requires supplementation (52). Rebound hypercalcemia between doses or after discontinuation of denosumab occurs frequently in children before skeletal maturity, despite attempts to prevent this with bisphosphonates or tapering of denosumab (19, 44-51, 53-55, 57, 59, 62-64, 67, 74-78). Of note, the Amgen-sponsored study of denosumab in children with osteogenesis imperfecta was stopped early because of the frequency of serious adverse events, most notably hypercalcemia (79).

This “rebound phenomenon” after offset of denosumab action has been described in both adults and children. A rapid decrease in areal bone mineral density (BMD) and increased risk of vertebral fractures has been observed 3 to 24 months after cessation of denosumab in adults treated for osteoporosis (80-84). This is associated with a rebound overshoot of bone turnover markers to above pretreatment levels, indicating significantly increased osteoclast formation and activity (85). These observations have led to the recommendation for a bisphosphonate to be administered 6 months after denosumab is discontinued in adults (86, 87). The rebound phenomenon is thought to occur because of accumulation of dormant osteoclast precursors during treatment that rapidly differentiate into osteoclasts once RANKL stimulation returns at the offset of denosumab action (88, 89). In mice, osteoclasts undergo fission and fusion and thus have the ability to regulate their own population in number and function and adapt quickly to changing situations (90). Such osteoclast fission occurs under stimulation with OPG, forming daughter cells termed “osteomorphs,” which rapidly refuse into active osteoclasts on OPG withdrawal (91). This process of “osteoclast recycling” is thought to contribute to the rebound phenomenon seen with denosumab treatment (89).

The rebound overshoot of bone turnover in children and adolescents before skeletal maturity appears to be proportionally greater than in adults and is associated with a greater risk of hypercalcemia (92). The pediatric predominance is hypothesized to be due to 1 or a combination of factors, including higher baseline bone turnover, the perpetual formation of new, RANKL-rich bone during growth, greater retention of metaphyseal bone during treatment, and more time for secondary mineralization throughout the skeleton (40). The rebound phenomenon can lead to severe hypercalcemia, which can cause acute kidney injury. Hypercalcemia usually requires hospitalization and occasionally intensive care admission (51, 53-55, 59). Severe rebound hypercalcemia invariably requires intervention with intravenous bisphosphonates or additional denosumab (92).

A recent systematic review identified a highly variable duration between the last dose of denosumab and onset of hypercalcemia (interquartile range, 3-5 months; range, 1-7 months) (92). The rebound phenomenon can also occur while children are receiving active denosumab therapy (77). For children started on monthly dosing regimens, it usually occurs when the interval between doses is extended, but for children on less frequent, lower dose regimens, it may occur when the interval is decreased (93), presumably once a certain threshold of antiresorption is reached.

**Figure 4.**
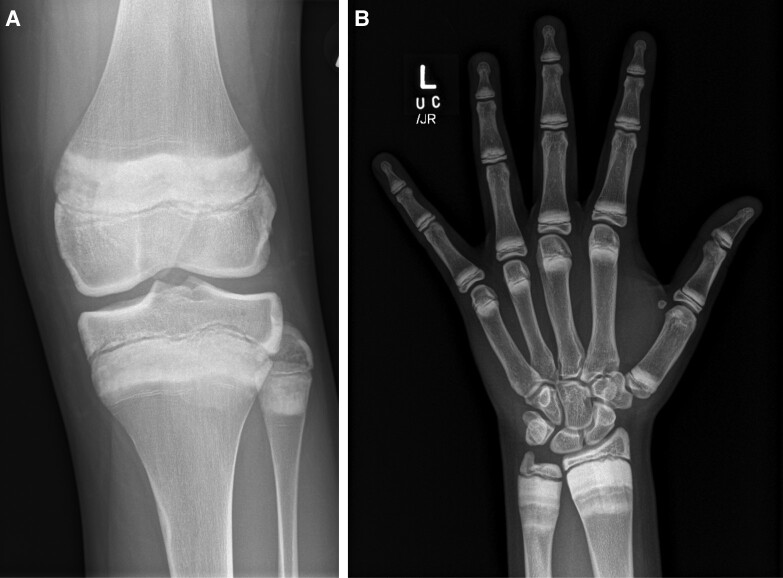
Metaphyseal sclerosis and undermodeling with early Erlenmeyer flask deformity in adolescent patients after denosumab therapy for (A) 18 months and (B) 12 months followed by a second course after a 6-month interval. Both patients also had bisphosphonates after denosumab therapy.

Denosumab therapy is systemic and therefore results in an increase in BMD, especially in young children and during rapid growth phases (2). Metaphyseal sclerosis occurs during treatment and modeling defects, similar to those seen after excessive doses of IV pamidronate (94), have been described (Fig. 4). It is not known what the biomechanical effect of antiresorption-mediated modeling defects may be in the long term. Stress fractures in areas of metaphyseal sclerosis rarely have been described (95). Experience suggests that linear growth is not impaired by denosumab therapy, similar to bisphosphonate therapy (96), but further data are needed. To date, nerve entrapment related to bone overgrowth, as seen in osteopetrosis (97, 98), has not been reported in children treated long term with denosumab.

The effect of denosumab on dental development has not been studied systematically, but a similar effect to bisphosphonate therapy is expected, including delayed dental age in children with osteogenesis imperfecta (99, 100). The risk of osteonecrosis of the jaw (ONJ) secondary to high-dose denosumab is substantially higher than that associated with bisphosphonates in adults (0.7%-5.0% vs 0.02%-0.05%, respectively) and risk correlates with total denosumab exposure (38, 101). ONJ has been reported in one 19-year-old adolescent treated with denosumab for GCTB and was associated with poor dental health and the need for a dental extraction (57).

## Recommendations

Children and adolescents with RANKL-mediated disorders may present to different pediatric specialists. As with any rare disease, patients need to be managed in a tertiary specialist center, where cross-disciplinary consultation is possible to ensure correct and rapid diagnosis and to avoid unnecessary interventions.

### Planning of Therapy

After confirming the diagnosis, an interdisciplinary team should be formed to manage a child or adolescent safely and effectively with denosumab therapy. This team should include a pediatric endocrinologist or specialist with expertise in managing denosumab treatment and the rebound phenomenon. It would typically also include a surgeon (orthopedic, ear nose and throat, maxillofacial, plastic/reconstructive, and/or neurosurgical, depending on the site of the target lesion/s), pathologist, radiologist, dentist and case coordinator. An oncologist and nuclear medicine physician are required for RANKL-mediated bone tumors.

The decision to initiate denosumab therapy should be made using a multidisciplinary approach and in discussion with the family. The aims and duration of treatment should be defined before commencement, and the risks and benefits weighed against surgery or other approaches. Lifelong treatment should be avoided wherever possible and this needs to be taken into account when considering denosumab for patients with congenital RANKL-mediated disorders, such as FD and JPD. Some RANKL-mediated bone tumors are expected to have a permanent response to a short course of denosumab, but many will require long-term therapy to maintain remission or, otherwise, subsequent surgery.

Factors that may increase the risks associated with denosumab therapy should be considered, including fractures and dental disease, increased bone turnover states, and medical conditions affecting mineral homeostasis. For bone tumors, surgical assessment is required to determine if any surgical management is required before denosumab, to ensure structural integrity of the bone involved or prevent spinal cord complication in cases involving the vertebrae.

Regular clinical review, laboratory tests, and adherence to calcium and vitamin D supplements are critical. The capacity of the family to adhere to the treatment regimen must be assessed before treatment begins. Treatment is usually coordinated in a tertiary children's hospital but may be facilitated in a regional hospital in a shared care setting.

### Therapy—Treatment Phase

Standard denosumab dosing for RANKL-mediated bone tumors in adults is 120 mg every 4 weeks with loading doses on days 8 and 15, as used for GCTB (38, 39). Most reported pediatric cases have received either the adult dosing or adjusted for body size (70 mg/m^2^ or 1.7 mg/kg) with the same loading regimen and then a 4-week interval (Table 1). However, there is currently no published pharmacological data to inform pediatric dosing (40). Most protocols have continued treatment for at least 12 months. Lower doses, from 0.5 to 1 mg/kg, and longer intervals have been used for other RANKL-mediated disorders (48, 67, 78).

**Table 1.** Denosumab doses and regimens used in published case reports and series

Dose and regimen	Indication	Duration	Patients	Citations
120 mg days 1, 8, 15, 29, then every 4 wk	CGCG	6-18 mo	5	(60, 64, 75, 76)
	ABC	6-24 mo	15	(53, 58)
	GCTB	8-43 mo	16	(38, 52, 57, 62, 63)
	JXG	3 mo	1	(19)
	FD (adults)	6 mo	8 adults	(27)
70 mg/m^2^ (maximum, 120 mg) days 1, 8, 15, 29, then every 4 wk	CGCG	12-18 mo	10	(45, 46)
	ABC	6-24 mo	22	(44, 51, 54, 59)
	GCTB	12 mo, then interval extended	1	(43)
1.7 mg/kg (maximum, 120 mg) days 1, 8, 15, 29, then every 4 wk	CGCG	6-25 mo	4	(49)
60 mg days 1, 8, 15, 29, then every 4 wk	ABC	9-17 mo	7	(50, 53)
	GCTB	14 mo	2	(50)
1-1.5 mg/kg monthly	FD	7 mo-3.5 y	2	(48, 78)
60 mg every 3 mo	FD (adults)	13-30 mo	12 adults	(32)
0.5 mg/kg	JPD	2 doses 7 wk apart	1	(67)

All cases are <20 years of age unless otherwise stated.

Abbreviations: ABC, aneurysmal bone cyst; CGCG, central giant cell granuloma; FD, fibrous dysplasia; GCTB, giant cell tumor of bone; JPD, juvenile Paget disease; JXG, juvenile xanthogranuloma.

Given the exquisite sensitivity of RANKL-mediated disorders to denosumab, a lower dose, longer interval, or shorter treatment duration may be preferred to reduce the risk of hypercalcemia after discontinuation. Short courses of 3 to 6 months have been effective in a few reported cases of ABC, although without adequate follow-up to assess risk of reactivation (58). In lesions at higher risk of reactivation after discontinuation, such as CGCG, gradual extension of the dose interval after a 6-month course is a strategy being explored to try to maintain lesion remission with less metaphyseal bone retention. An alternative approach for focal lesions is to give a 6-month course followed by a break in treatment, which will allow resorption of sclerotic bone, with repeated courses as needed if lesions become active again.

Oral calcium and cholecalciferol supplementation are necessary during denosumab therapy. Elemental calcium 500 mg daily and cholecalciferol 1000 IU daily are recommended for adults, but adolescents have higher daily calcium requirements and most have required higher doses for some periods during denosumab therapy (44, 45). The dose should be adjusted based on serum calcium levels. Some experts prescribe supplements continuously throughout the treatment period, others only for a week after each denosumab dose. Serum 25-hydroxyvitamin D levels should be ≥50 nmol/L (20 ng/mL) before commencement of denosumab and maintained throughout the period of therapy. Calcitriol or alfacalcidol may be helpful in preventing or managing hypocalcemia, especially in the early weeks of treatment and in patients with FD who may have intrinsically suppressed 1,25(OH)_2_-vitamin D because of increased fibroblast growth factor-23 production. Phosphate supplementation may be required if hypophosphatemia occurs; however, clinicians should be conscious of the potential for phosphate supplements to inhibit intestinal calcium absorption.

### Monitoring—Efficacy

Regular imaging of focal lesions is required during and after treatment with intervals individualized in discussion with the surgeon and radiologist, depending on the type and location of the lesion(s). Magnetic resonance imaging (MRI) can measure lesion dimensions and analyze the soft-tissue component, with reduced signal intensity as a marker of treatment response (102, 103). Computed tomography (CT) allows monitoring of lesion ossification and dimensions and may assist surgeons to assess the structural integrity of the bone containing the lesion, especially when in critical locations, such as the spine (102). Either or both modalities are recommended at baseline and every 6 months during treatment and for 2 years after discontinuation, with reduced frequency thereafter if appropriate. Plain radiographs may be used in addition or instead, especially if more frequent monitoring is desired (eg, every 3 months). Radiographs will demonstrate progressive intralesional osteosclerosis, particularly at the margins, during treatment (102), and posttreatment reactivation may be detected as a new internal lucency.

Positron emission tomography/computed tomography (PET/CT) is sensitive for monitoring the metabolic response of lesions and allows early detection of reactivation (45, 102, 104-106). The low-dose CT component may also be used to monitor lesion dimensions and ossification in place of a standard-dose CT if the higher resolution is not required. Fluorodeoxyglucose PET/CT is used for bone tumors and may be performed at baseline and after 3 to 6 months to confirm treatment response, then repeated every 6 months until at least 2 years after discontinuation of denosumab to monitor for reactivation. ^18^F-sodium fluoride PET/CT is the imaging modality of choice for FD and can be performed at 6- to 12-month intervals, depending on the planned treatment course (27, 107).

The skeletal changes of JPD in response to denosumab are unknown as treatment in the single case report could not be continued long enough to demonstrate these (67). Further research will be required to determine appropriate monitoring.

### Monitoring—Safety

Serum calcium and phosphate monitoring during and after denosumab therapy is essential. The highest risk of hypocalcemia is in the first week of treatment. Serum calcium should be monitored weekly until maintained in the normal range, or for the first month if loading doses are given. This is often reduced to monthly thereafter, but frequency should be adjusted based on calcium levels. Hypophosphatemia can occur at any time during treatment.

We recommend using dual-energy x-ray absorptiometry (DXA) to monitor BMD, especially in cases that may require long-term denosumab. BMD increases during denosumab therapy and adjustment of dose or frequency may be required if height-adjusted total body less head or lumbar spine BMD *Z*-score exceeds +2.0. Sclerosis of denosumab-targeted lesions, especially when in the lumbar spine, may influence BMD measurement; this should be taken into consideration when interpreting results. A common approach is to measure BMD at baseline and annually during treatment, and annually for at least 2 years after discontinuation to monitor rebound loss of bone mass. Because of reports of vertebral compression fractures in adults 6 to 12 months after discontinuation of denosumab (84), some centers are assessing vertebral shape using plain radiographs or DXA-based “vertebral fracture assessment” in this period. In those requiring long-term or multiple courses of denosumab, ophthalmology and audiology assessments may be warranted to monitor for the theoretical risk of cranial nerve compression secondary to hyperostosis.

Dental assessment should be arranged prior to commencing denosumab so that any dental issues can be addressed before treatment to minimize the risk of ONJ, and dental review should continue every 6 months during treatment. Where possible, elective dental procedures should either be completed before starting or delayed until after completion of denosumab treatment.

Renal tract ultrasound should be performed at baseline and 6 to 12 months after discontinuation of denosumab, or annually during treatment if the denosumab interval is >1 month, to screen for nephrocalcinosis as a consequence of the rebound phenomenon.

Growth should be monitored during and after denosumab treatment. Knee and wrist radiographs will demonstrate metaphyseal sclerosis and may be useful to monitor for modeling defects.

### Prevention and Management of “Rebound” Hypercalcemia

The primary safety concern in children and adolescents before skeletal maturity is the prevention and early detection of hypercalcemia between denosumab doses or after discontinuation. Skeletally mature adolescents appear to be at lower risk, but still require close monitoring. The aim of therapeutic strategies to prevent rebound hypercalcemia is to allow gradual resorption of excess bone accumulated during treatment while preventing resorption rapid enough to exceed renal calcium excretion capacity, which leads to hypercalcemia. Hydration is an important determinant of renal calcium excretion and the authors have found it helpful to prescribe a daily oral fluid target to ensure fluid intake meets or exceeds recommendations for age and body size, with adjustments for high temperatures and physical exercise.

IV bisphosphonates are invariably needed to prevent rebound hypercalcemia after denosumab discontinuation or during tapering of the dose or interval. The premise is to use the longer duration of action and less potent antiresorptive effect of bisphosphonates to decrease the rapid rebound of bone turnover that occurs at denosumab offset. A number of approaches have been used and there are currently insufficient data to recommend 1 over the others:

- Zoledronate 0.025 mg/kg 4 and 8 weeks after the last denosumab dose, followed by risedronate 35 mg weekly (or every 2 weeks if weight <30 kg (108)) for 6 months. If hypercalcemia occurs, an additional dose of zoledronate 0.025 mg/kg is given. Alendronate may be used instead of risedronate and dose or frequency could be increased if biochemistry suggests impending hypercalcemia.
- Zoledronate 0.05 mg/kg before the first dose of denosumab and continued every 6 months throughout the treatment period. If hypercalcemia occurs during or after the treatment period, an additional dose of zoledronate 0.05 mg/kg is given. If hypercalcemia persists, denosumab 0.25 mg/kg is given.
- A lower/less-frequent dose denosumab regimen (which has been described to treat non-RANKL-mediated conditions) is denosumab 1 mg/kg alternating every 3 months with zoledronate 0.025 mg/kg (93).

If deescalation of therapy is desired to maintain a therapeutic effect but reduce the effect on BMD, denosumab therapy may be tapered by gradually extending the dose interval with the addition of a bisphosphonate. The following approach is in current use:

- Once the period of 4-weekly denosumab is completed, denosumab alternates with zoledronate 0.025 mg/kg every 4 weeks, and later every 6 weeks. A final zoledronate dose is given 12 weeks after the last denosumab dose. If hypercalcemia occurs at any time, an extra dose of zoledronate 0.025 mg/kg is given.

In the authors’ experience, tapering of the denosumab dose or interval without the use of an IV bisphosphonate is insufficient to prevent rebound hypercalcemia.

Serum calcium, PTH and urinary calcium/creatinine ratio on fasting second-void urine sample should be closely monitored during denosumab treatment and for at least 8 months following discontinuation. Measurement should occur at least monthly for this full period, but increased frequency is often warranted at times of higher risk, including when the dose interval is extended and between 2 and 6 months after discontinuation. Patients and families should be thoroughly counselled to recognize symptoms of hypercalcemia, which should prompt measurement of serum calcium. It is the authors’ experience that hypercalcemia is a late, abrupt manifestation of the rebound phenomenon. Further zoledronate at 0.025 to 0.05 mg/kg or denosumab is usually required to manage hypercalcemia, but caution is required with bisphosphonates if there is acute kidney injury. Calcium monitoring should then continue for a further 6 months because hypercalcemia may recur as the antiresorptive effect declines again.

Suppressed PTH and elevated bone turnover markers and urine calcium excretion are early markers of the rebound phenomenon that precede hypercalcemia and may assist in identifying impending hypercalcemia (57, 59, 77, 93). Many experts evaluate the trajectories of these biochemical indices to guide therapeutic approaches to prevent hypercalcemia. Experience suggests that rapidly rising urinary calcium/creatinine and/or bone turnover markers, or posttreatment serum C-terminal telopeptide of type 1 collagen level above the pretreatment value, signal a high-risk period for hypercalcemia.

## Prospective Data Collection

Ongoing international collaboration and data sharing are needed to refine the approach to denosumab use in pediatrics to improve safety and efficacy. The minimum data set shown in Table 2 is proposed for collection by individual centers with the aim of future collation and analysis to inform improved evidence-based recommendations.

**Table 2.** Proposed minimum data set for collection by individual centers

Data points	Frequency
*Efficacy data*	
Surgical intervention − Timing, procedure, outcome	
Imaging of focal lesions − CT or MRI: lesion dimensions and calculated volume − CT: radiodensity (Hounsfield units) − PET: tracer-uptake (SUVmax) − Radiograph: longest diameter	Modality and timing will depend on the disease being treated and availability in treating centers. Nevertheless, radiographic measures of treatment response and duration of response should be documented, including after discontinuation. For bone tumors, suggest minimum every 6 months during treatment and for 2 years after discontinuation, less frequently thereafter.
*Safety data*	
Fasting serum calcium, phosphorus, alkaline phosphatase, PTH, CTX, P1NP Urine calcium/creatinine ratio (second void, fasting)	Baseline At least monthly for 3 months after treatment commencement or any dose/interval change At least every 3 months for remainder of treatment Every 2-4 weeks for 8 months after discontinuation
Height and weight	Baseline and every 3-6 months thereafter
25-hydroxyvitamin D	Baseline and every 6 months thereafter
Bone age radiograph	Baseline and annually thereafter
BMD using DXA as per ISCD official positions (109)	Baseline and annually during treatment At end of treatment and annually for at least 2 years
Dental assessment − Tooth eruption, retention − Caries − Orthodontic treatment − Osteonecrosis of the jaw	Baseline and every 6 months thereafter
Vertebral fracture assessment by DXA or plain radiograph	6 and 12 months after discontinuation of denosumab
Nephrocalcinosis on ultrasound	All results during treatment if performed 6-12 months after discontinuation of denosumab

Abbreviations: BMD, bone mineral density; CT, computed tomography; CTX, C-terminal telopeptide of type 1 collagen; DXA, dual-energy x-ray absorptiometry; ISCD, International Society for Clinical Densitometry; MRI, magnetic resonance imaging; PET, positron emission tomography; P1NP, procollagen 1 intact N-terminal pro-peptide; SUVmax, maximum standardized uptake value.

## Future Directions and Research Questions

The use of denosumab in children is still in its early days and there is much yet to be learned. Further research is needed to answer the following important questions (and, in some cases, is already under way):

- What are the optimal denosumab treatment regimens to treat the different RANKL-mediated bone disorders?
- Can a lower dose of denosumab achieve similar efficacy with reduced risk of complications?
- What is the effect of denosumab on skeletal modeling and remodeling, linear growth, dental development, and risk of ONJ?
- Is there an increased risk of fracture at metaphyseal sclerosis lines during or following denosumab therapy?
- What is the optimal regimen for denosumab, with or without bisphosphonate, and monitoring to prevent rebound hypercalcemia?
- For which bone tumors can a single course of denosumab be expected to induce long-term remission and what is the least-intensive treatment regimen that will achieve this?
- For RANKL-mediated bone tumors that reactivate after discontinuation of denosumab
  - How can denosumab best be used as an adjuvant/neoadjuvant to surgery?
  - What is the optimal treatment regimen to achieve and maintain long-term tumor control while minimizing the risk of complications?
- How can denosumab be used safely long-term in congenital RANKL-mediated disorders to maintain a therapeutic effect without a substantial risk of complications?

Given the rarity of RANKL-mediated disorders in childhood, these questions will necessarily be addressed through ongoing international dialogue and collaboration in order to develop the level of evidence required for a more detailed consensus statement.

## Data Availability

Data sharing is not applicable to this article as no datasets were generated or analyzed during the current study.

## Abbreviations

ABC: aneurysmal bone cyst
BMD: bone mineral density
CGCG: central giant cell granuloma
CT: computed tomography
DXA: dual-energy x-ray absorptiometry
FD: fibrous dysplasia
GCTB: giant cell tumor of bone
JPD: juvenile Paget disease
MRI: magnetic resonance imaging
ONJ: osteonecrosis of the jaw
OPG: osteoprotegerin
PET: positron emission tomography
RANKL: receptor activator of nuclear factor kappa-B ligand
